# High-Resolution Magic Angle Spinning Metabolomic Profiling of IDH-Wild-Type Glioblastoma Reveals a Composite Surgical Sampling Signature Shaped by Clinical and Anatomical Tumor Features

**DOI:** 10.3390/metabo16050296

**Published:** 2026-04-27

**Authors:** Julien Todeschi, Caroline Bund, Hassiba Outilaft, Hélène Cebula, Izzie-Jacques Namer

**Affiliations:** 1 Service de Neurochirurgie, Hôpitaux Universitaires de Strasbourg (HUS), 67200 Strasbourg, France; julien.todeschi@chru-strasbourg.fr (J.T.); helene.cebula@chru-strasbourg.fr (H.C.); 2 ICube, Université de Strasbourg/CNRS (UMR 7357), 67085 Strasbourg, France; c.bund@institut-strauss.fr (C.B.); h.outilaft@institut-strauss.fr (H.O.); 3 Service de Médecine Nucléaire et d’Imagerie Moléculaire, Institut Strauss, 67200 Strasbourg, France

**Keywords:** glioblastoma, HRMAS NMR, metabolomics, surgical sampling, intratumoral heterogeneity, biopsy, resection, IDH-wild-type

## Abstract

Background/Objectives: Tissue-based metabolomic readouts in IDH-wild-type glioblastoma may be strongly shaped by how tumor tissue is surgically accessed and sampled. We aimed to determine whether, and to what extent, surgical sampling context structures the HRMAS metabolic landscape, and to disentangle sampling-related contributions from clinico-anatomical confounders. Methods: We retrospectively analyzed 99 patients with de novo IDH-wild-type glioblastoma (35 biopsy-only, 64 resection: 40 gross-total, 21 near-total, 3 subtotal), yielding 166 HRMAS spectra and 47 quantified metabolites (nmol/mg). Patient-level profiles were compared using PCA, metabolite-wise testing, pathway-level aggregation (10 pathways), and variance partitioning by PERMANOVA, both unadjusted and adjusted for age, WHO PS, deep-seated location, midline involvement, multifocality, MGMT methylation, and eloquent area. Sensitivity analyses included clinico-anatomically restricted subgroups, 15 canonical metabolite ratios, and Probabilistic Quotient Normalization. Intratumoral heterogeneity was assessed in 44 multi-sampled patients. Results: Biopsy-only and resection-derived cases separated along PC1 in unsupervised PCA (62.6% variance; *p* < 0.001), with 42/47 metabolites differing after FDR correction. However, the surgical group explained only 2.6% of the total variance (PERMANOVA *p* = 0.026), and this share was no longer significant after confounder adjustment (*p* = 0.39). Clinico-anatomical restriction progressively attenuated the effect (42/47 → 1/47 significant metabolites). Ratio-based and PQN analyses showed a residual compositional difference beyond scaling (13/15 ratios; 16/47 metabolites). Intratumoral heterogeneity was greater in resections and preserved in an n-matched analysis (*p* = 0.020). Conclusions: The apparent biopsy-versus-resection metabolic difference is largely a composite signal reflecting clinico-anatomical patient selection with a smaller tissue-composition contribution. Biopsy-only and resection-derived specimens should not be pooled uncritically in tissue-based metabolomic studies of glioblastoma.

## 1. Introduction

Glioblastoma, IDH-wild-type, remains the most aggressive primary brain tumor in adults and is increasingly recognized as a highly dynamic disease characterized by marked cellular, spatial, and metabolic plasticity. In this setting, tumor metabolism is not merely a downstream consequence of oncogenic signaling, but an integrated functional readout of tumor cell states evolving within a microenvironment shaped by hypoxia, nutrient stress, vascular remodeling, and diffuse invasion. Metabolomic profiling therefore offers a particularly relevant framework for capturing biologically meaningful tumor states that may not be fully resolved by histopathology or genomics alone [1,2].

High-resolution magic-angle-spinning nuclear magnetic resonance (HRMAS NMR) spectroscopy is particularly well suited to brain tumor research, as it provides metabolic information directly from intact tissue while preserving tissue architecture for subsequent pathological correlation. Early landmark studies demonstrated that ex vivo HRMAS reveals substantial metabolic microheterogeneity within individual glioblastomas, establishing this technique as a relevant tool for investigating tumor organization at clinically meaningful spatial scales [3,4,5]. More recent image-guided HRMAS work has further shown that tissue-level metabolic profiles correlate with specific histopathological features, including mitotic activity, axonal disruption, and vascular proliferation [6].

Yet the biological promise of glioblastoma metabolomics is inseparable from a major methodological challenge: what is measured depends critically on what is sampled. Glioblastoma is not a metabolically uniform lesion. The enhancing core, infiltrative margin, perinecrotic territories, and invasive edge may harbor distinct metabolic programs shaped by differences in cellular density, vascular supply, oxygenation, stromal interactions, and local selective pressures. Recent metabolomic studies continue to support the existence of substantial intratumoral metabolic heterogeneity and region-specific metabolic signatures in glioblastoma. However, tissue-based metabolomic analyses are often performed on specimens obtained under markedly different surgical conditions, ranging from stereotactic biopsy to resection-derived sampling, although these approaches are unlikely to interrogate biologically equivalent tumor compartments [2,7,8].

This distinction is not trivial. By design, stereotactic biopsy samples a narrow and clinically constrained region of the tumor, typically in lesions that are deep-seated, functionally eloquent, multifocal, or otherwise poorly amenable to resection. In contrast, resection-derived tissue is obtained from a broader and potentially more spatially diverse operative field. As a result, differences attributed to glioblastoma metabolism may partly reflect differences in surgical sampling context, tissue composition, and accessible tumor territory rather than intrinsic biological divergence alone. In other words, surgical sampling itself may represent a major source of structured metabolic variation. Clarifying this issue is essential not only for biological interpretation but also for the development of robust tissue-based biomarkers and for any attempt to quantify intratumoral metabolic heterogeneity across patients.

Accordingly, the primary objective of the present study was to determine whether the surgical sampling context is a major determinant of the HRMAS metabolic landscape in newly diagnosed IDH-wild-type glioblastoma. We first compared patient-level metabolic profiles between biopsy-only and resection-derived cases to assess whether sampling context exerted a major structuring effect on the observed metabolome. We then examined this effect at both the individual metabolite and pathway levels using biologically defined metabolic groupings. Finally, in multi-sampled tumors, we quantified intratumoral metabolic heterogeneity using a distance-based approach derived from spectrum-level profiles. By combining ex vivo HRMAS profiling with a clinically grounded surgical dichotomy, our aim was to better distinguish sampling-related metabolic variation from tumor-intrinsic heterogeneity and to clarify the interpretative limits of tissue-based metabolomics in glioblastoma.

## 2. Materials and Methods

### 2.1. Study Design and Patient Population

This single-center study was based on a retrospective analysis of prospectively collected tumor specimens and associated clinical data from the institutional HRMAS NMR tissue collection program at Strasbourg University Hospitals (Hôpitaux Universitaires de Strasbourg, France). Adult patients undergoing a first surgical procedure (biopsy or resection) for newly diagnosed glioblastoma between January 2019 and December 2024 were screened. Inclusion criteria were: (i) histopathologically confirmed glioblastoma, CNS WHO grade 4; (ii) IDH-wild-type status established by routine integrated histomolecular assessment; and (iii) availability of at least one interpretable HRMAS spectrum from the initial surgical episode. Patients with IDH-mutant diffuse gliomas, specimens obtained from recurrent tumors rather than from the de novo setting, prior glioma-directed treatment, or non-interpretable HRMAS spectra were excluded. According to the surgical context, patients were classified as biopsy-only or resection cases.

The extent of resection was determined by visual and volumetric comparison of postoperative early (≤72 h) contrast-enhanced T1-weighted MRI with the preoperative MRI, in accordance with the RANO Resect group criteria [9]. Resections were classified as: gross-total resection (GTR), no residual contrast-enhancing tumor; near-total resection (NTR), residual contrast-enhancing tumor < 1 cm^3^; and subtotal resection (STR), residual contrast-enhancing tumor ≥ 1 cm^3^ but with operative intent of maximal debulking. Biopsy-only procedures comprised diagnostic stereotactic sampling without attempt at tumor resection.

### 2.2. Tissue Sampling and HRMAS NMR Acquisition

Tumor specimens were collected with minimal ischemic delay after surgery, immediately snap-frozen in liquid nitrogen, and stored at −80 °C until analysis, according to the institutional HRMAS workflow previously used in Strasbourg HRMAS studies [10,11,12], with identical post-sampling pre-analytical handling in both biopsy-only and resection cases.

In biopsy-only cases, stereotactic sampling was performed using the ROSA robotic platform (Zimmer Biomet, Warsaw, IN, USA), whereas in resection cases, tissue sampling was performed under neuronavigation guidance. Samples were obtained either from the contrast-enhancing tumor or from the immediate tumoral FLAIR periphery, while remaining within the tumoral area. Structured per-spectrum annotation of contrast-enhancing versus FLAIR localization was not systematically recorded in our database across the entire 2019–2024 window; accordingly, a fully rigorous within-region comparison could not be performed retrospectively. A postoperative day-1 CT scan was routinely performed as part of standard postoperative control.

For HRMAS analysis, approximately 15–20 mg of frozen tissue was placed into a disposable 25 μL KelF insert with 5 μL of deuterium oxide (D_2_O) for field-frequency locking. Shortly before HRMAS analysis, the insert was placed into a standard 4 mm ZrO_2_ rotor. Spectra were acquired on a Bruker Avance III 500 spectrometer operating at a proton frequency of 500.13 MHz, equipped with a triple-resonance gradient HRMAS probe (^1^H, ^13^C, ^31^P), at 4 °C, using a one-dimensional ^1^H CPMG (Carr–Purcell–Meiboom–Gill) sequence (interpulse delay of 286 μs, 328 loops, effective CPMG filter length of 93 ms, acquisition time of 10 min). Chemical shift was calibrated to the methyl resonance of L-lactate at 1.33 ppm. When required, two-dimensional ^1^H-^13^C HSQC (Heteronuclear Single Quantum Correlation) experiments were performed to confirm metabolite assignments [12].

The HRMAS acquisition pipeline was stable across the study window (2019–2024). The same 500 MHz Bruker Avance III spectrometer, the same triple-resonance gradient HRMAS probe, the same 4 °C CPMG acquisition protocol, the same Chenomx-based quantification pipeline, and the same reference metabolite database were used throughout, and the same primary HRMAS operator handled spectral acquisition and processing across the entire study period. In addition, each spectrum was visually inspected for quality before metabolite quantification, and any spectrum that failed internal quality thresholds was excluded at the screening stage rather than retained as poor-quality data.

HRMAS spectral acquisition, processing, and metabolite quantification were performed in a blinded manner with respect to the surgical sampling context. Metabolite assignment was carried out using the customized database of the Strasbourg metabolomics platform, whereas quantification was performed using Chenomx NMR Suite^®^ (Chenomx Inc., Edmonton, AB, Canada). This database, which includes approximately 76 metabolites, is described in detail elsewhere [13].

Each HRMAS specimen was weighed before spectral acquisition, and metabolite concentrations were determined using a quantification method based on a DSS signal calibrated from alanine, then adjusted using the ERETIC signal, and subsequently normalized to tissue mass and expressed in nmol/mg. The same mass-based normalization pipeline was applied uniformly across both groups, and the recorded tissue mass distributions were comparable between groups (biopsy median 16.2 mg, interquartile range 11.8–19.1; resection median 16.65 mg, interquartile range 14.9–19.0; Mann–Whitney *p* = 0.13).

### 2.3. Metabolite Quantification and Construction of Analytic Datasets

The 47-metabolite panel used in the present analysis was derived from the full Strasbourg HRMAS reference database (approximately 76 assignable metabolites) [13] through the following stepwise quality-control and harmonization procedure: (i) exclusion of metabolites requiring experiment-specific two-dimensional confirmation not performed systematically in the retrospective cohort; (ii) exclusion of metabolites with unresolvable peak overlap at 500 MHz in tumor tissue spectra; (iii) exclusion of metabolites detected in fewer than 10% of spectra; (iv) harmonization of metabolite names and quantification units across the 2019–2024 analytic window; (v) retention of the 47 metabolites meeting all the above criteria.

Some patients contributed more than one intratumoral sample, reflecting multi-sampling within the contrast-enhancing tumor and/or the immediate tumoral FLAIR periphery during the same surgical episode. For patients with more than one available spectrum, a patient-level metabolic profile was generated by calculating the median value of each metabolite across spectra. These patient-level profiles were used for all between-group comparisons, principal component analysis, pathway-level analyses, and exploratory survival analyses.

To complement metabolite-level analyses, 41 assigned metabolites were grouped into 10 biologically defined metabolic pathways, while 6 metabolites remained unassigned and were excluded from pathway-score calculations (Table S2). The 10 pathways and their constituent metabolites were: (1) Glycolysis (lactate, glucose, alanine, glycerol, 3-hydroxybutyrate, acetate); (2) Glutaminolysis (glutamate, glutamine, asparagine, aspartate, proline, 2-hydroxyglutarate); (3) Neuronal markers (N-acetylaspartate, GABA, adenosine, O-acetylcholine); (4) Creatine/energy buffering (creatine, phosphocreatine); (5) Membrane phospholipid metabolism (choline, phosphocholine, glycerophosphocholine, ethanolamine); (6) TCA cycle (succinate, fumarate); (7) One-carbon/methylation metabolism (glycine, serine, methionine, formate, threonine, betaine); (8) Redox and antioxidants (glutathione, ascorbate, hypotaurine, taurine, allocystathionine); (9) Osmolytes (myo-inositol, scyllo-inositol, ethanol); (10) Branched-chain amino acids (leucine, isoleucine, valine). The six remaining metabolites (arginine, lysine, N-acetyl-lysine, ornithine, phenylalanine, tyrosine) could not be reliably assigned to a single biological pathway at the resolution of the present dataset and were therefore excluded from pathway-score calculations (Table S2). For each patient, a pathway score was computed as the mean z-scored metabolite concentration for the corresponding pathway.

Intratumoral metabolic heterogeneity was assessed only in patients with at least two available spectra from the same surgical episode. For these patients, pairwise Euclidean distances were calculated from z-scored spectrum-level metabolite profiles, and a patient-level heterogeneity score was defined as the mean pairwise Euclidean distance across spectra from the same patient.

### 2.4. Clinical Variables and Endpoints

Clinical, radiological, and molecular variables were extracted from electronic medical records and preoperative imaging, including age at surgery, sex, WHO performance status, tumor side, deep-seated location, eloquent area involvement, midline/corpus callosum/deep nuclei extension, multifocal presentation, dominant hemisphere involvement, maximum contrast-enhancing tumor diameter, MGMT promoter methylation status, extent of resection, and administration of the Stupp regimen. Overall survival was defined as the time from the first surgery to death from any cause or to the last follow-up.

### 2.5. Ethics

The study was conducted in accordance with the Declaration of Helsinki and approved by the Ethics Committee of Strasbourg (approval nos. 2003-100, 12 September 2003; 2013-37, 11 December 2013; and IRB-2025-14). Written informed consent was obtained from all patients for tissue banking and for the research use of associated clinical data.

### 2.6. Statistical Analysis

Continuous variables are reported as median [interquartile range] and categorical variables as count (percentage), compared between biopsy-only and resection-derived groups using the Mann–Whitney U, Fisher’s exact, or chi-square test as appropriate. For metabolite-wise comparisons, the Mann–Whitney U test was applied to patient-level profiles, fold changes were calculated from group medians (with a pseudocount when at least one median was 0), and multiple testing was controlled using the Benjamini–Hochberg false discovery rate (FDR) procedure with FDR < 0.05 considered significant. Principal component analysis and pathway-level analyses (10 predefined pathway scores computed as the mean z-score of constituent metabolites; see Section 2.3) were performed on z-scored patient-level profiles, with the same Mann–Whitney U/Benjamini–Hochberg framework applied to pathway-level comparisons.

Variance partitioning was performed using permutational multivariate analysis of variance (PERMANOVA) on the Euclidean distance matrix of z-scored patient-level profiles (9999 permutations). In the adjusted model, covariates (age, WHO performance status ≥ 2, deep-seated location, midline/corpus callosum/deep grey nuclei involvement, multifocality, MGMT promoter methylation, eloquent area) were partialled out by sequential linear residualization before recomputation of the distance matrix. Sensitivity PERMANOVA was repeated in subgroups restricted to non-multifocal tumors, tumors sparing the midline/deep grey nuclei, and their intersection.

To test whether the biopsy-versus-resection difference was driven by global tissue-scaling, two complementary strategies were applied. First, 15 canonical HRMAS-compatible metabolite ratios normalized to creatine, total-creatine, total-choline, or N-acetylaspartate were compared between groups (Mann–Whitney U with Benjamini–Hochberg correction), consistent with established HRMAS conventions in brain tumors [14]. Second, Probabilistic Quotient Normalization (PQN) was applied to the metabolite matrix—per-spectrum dilution factors were computed as the median of sample-to-reference ratios after integral normalization—and the full downstream pipeline (metabolite-wise comparisons, PCA, PERMANOVA) was re-run on the PQN-normalized data; dilution factors were also compared between groups.

Intratumoral metabolic heterogeneity was assessed in patients with ≥2 spectra using mean pairwise Euclidean distances on z-scored spectrum-level profiles, and differences between groups were assessed with the Mann–Whitney U test. Two sensitivity analyses addressed a potential confounding effect of the number of spectra per patient: (i) Spearman’s ρ between the heterogeneity score and n_spectra overall and within groups, and (ii) an n-matched comparison restricted to patients with exactly two spectra.

Exploratory survival analyses used univariable Cox proportional hazards models for each metabolite (z-scored, hazard ratios per +1 SD, Benjamini–Hochberg correction), and a multivariable Cox model including age and MGMT promoter methylation as a clinical validity check for overall survival. All tests were two-sided. Analyses were performed in Python 3.12.3 (NumPy v2.4.4, pandas v3.0.2, SciPy v1.17.1, statsmodels, lifelines, scikit-learn v1.8.0, Matplotlib v3.10.8, Seaborn v0.13.2).

## 3. Results

### 3.1. Study Flow and Analytic Cohorts

**Figure 1:**
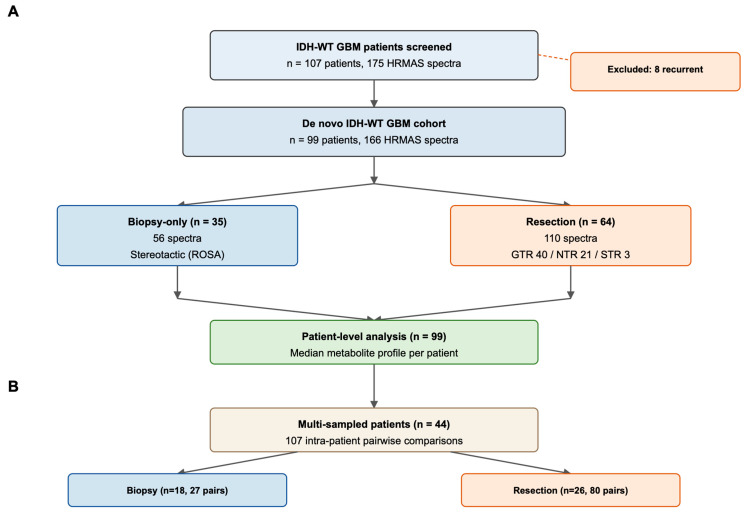
Study flowchart. (**A**) Of 107 patients with IDH-wild-type glioblastoma and available HRMAS spectra, 8 were excluded because the analyzed specimen corresponded to a tumor recurrence rather than the initial diagnosis, yielding a de novo cohort of 99 patients (166 spectra). Patients were classified as biopsy-only (n = 35; stereotactic sampling using the ROSA robotic platform, Zimmer Biomet, Warsaw, IN, USA) or resection (n = 64; including 40 gross-total, 21 near-total, and 3 subtotal resections performed under neuronavigation guidance). For each patient, when multiple spectra were available, patient-level metabolic profiles were computed as the median concentration across spectra. (**B**) Intratumoral metabolic heterogeneity was assessed in the 44 patients with at least two HRMAS spectra from the same surgical episode, yielding 107 intra-patient pairwise comparisons.

Among 107 patients with IDH-wild-type glioblastoma and available HRMAS spectra, 8 were excluded because the analyzed specimen corresponded to a tumor recurrence rather than the initial diagnosis. The final de novo cohort, therefore, comprised 99 patients and 166 HRMAS spectra. According to the surgical context, 35 patients were classified as biopsy-only and 64 as resection cases. Within the resection group, 40 underwent gross-total resection, 21 underwent near-total resection, and 3 underwent subtotal resection (Figure 1). For patient-level analyses, when multiple spectra were available for a given patient, metabolite values were aggregated to the median across spectra. Intratumoral metabolic heterogeneity was evaluated in the 44 patients with at least 2 HRMAS spectra obtained during the same surgical episode, yielding 107 intra-patient pairwise comparisons (Figure 1).

### 3.2. Clinical and Tumor Characteristics According to Surgical Sampling Group

Baseline clinical and tumor characteristics are summarized in Table 1. Age was comparable between biopsy-only and resection groups (63.5 [56.5–69.5] vs. 63.8 [55.9–69.0] years, *p* = 0.626), as was the proportion of male patients (60.0% vs. 70.3%, *p* = 0.373). By contrast, biopsy-only tumors were associated with a less favorable clinical and anatomical profile, with more frequent WHO performance status ≥ 2 (37.1% vs. 14.1%, *p* = 0.012), deep-seated location (85.7% vs. 17.2%, *p* < 0.001), eloquent area involvement (91.4% vs. 40.6%, *p* < 0.001), midline/corpus callosum/deep nuclei extension (62.9% vs. 3.1%, *p* < 0.001), multifocal presentation (60.0% vs. 9.4%, *p* < 0.001), and dominant hemisphere involvement (71.4% vs. 45.3%, *p* = 0.020). Bilateral tumors were observed only in the biopsy-only group. MGMT promoter methylation status did not differ between groups among patients with available data (48.5% vs. 48.3%, *p* = 1.000). As expected, the Stupp regimen was administered less frequently in biopsy-only patients than in resection patients (68.6% vs. 92.2%, *p* = 0.004). Overall survival was significantly shorter in the biopsy-only group (266 [79–509] days vs. 620 [360–951] days, *p* < 0.001) (Table 1). In an additional multivariable Cox model including age and MGMT promoter methylation status (n = 93; 78 events), both variables were independently associated with overall survival (age: HR 1.03 per year, 95% CI 1.00–1.06, *p* = 0.035; MGMT methylation: HR 0.55, 95% CI 0.34–0.89, *p* = 0.015), consistent with established prognostic factors.

### 3.3. Unsupervised Metabolic Structure Reveals a Structured Sampling-Related Axis

**Figure 2:**
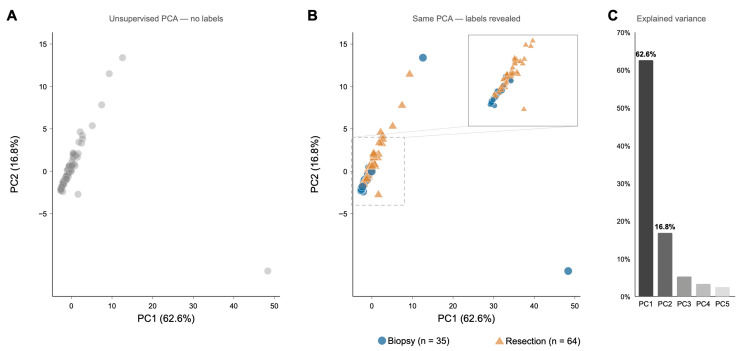
Unsupervised principal component analysis (PCA) of patient-level HRMAS metabolic profiles (47 metabolites, n = 99 de novo IDH-wild-type glioblastoma patients). (**A**) PCA score plot showing all patients in gray, demonstrating that the dominant axis of metabolic variation emerges without knowledge of the surgical context. (**B**) Same PCA score plot with group labels revealed post-hoc: biopsy-only (blue circles, n = 35) and resection (orange triangles, n = 64). The inset shows a magnified view of the main cluster (dashed rectangle), highlighting the overlap zone between groups. PC1 scores differed significantly between groups (Mann–Whitney U, *p* < 0.001). (**C**) Scree plot showing the proportion of variance explained by each principal component; PC1 alone captured 62.6% of total metabolic variance.

Principal component analysis of patient-level HRMAS metabolic profiles, based on 47 quantified metabolites, showed that the dominant axis of metabolic variation emerged in an unsupervised manner (Figure 2A). When surgical group labels were overlaid post hoc, biopsy-only and resection cases separated predominantly along the first principal component (Figure 2B). PC1 scores differed significantly between groups (Mann–Whitney U, *p* < 0.001), and PC1 alone accounted for 62.6% of the total variance, indicating that surgical sampling context captured the major source of variation in the dataset (Figure 2C).

### 3.4. Metabolite-Level Comparison Shows a Broad Shift Toward Higher Levels in Resection-Derived Specimens

**Figure 3:**
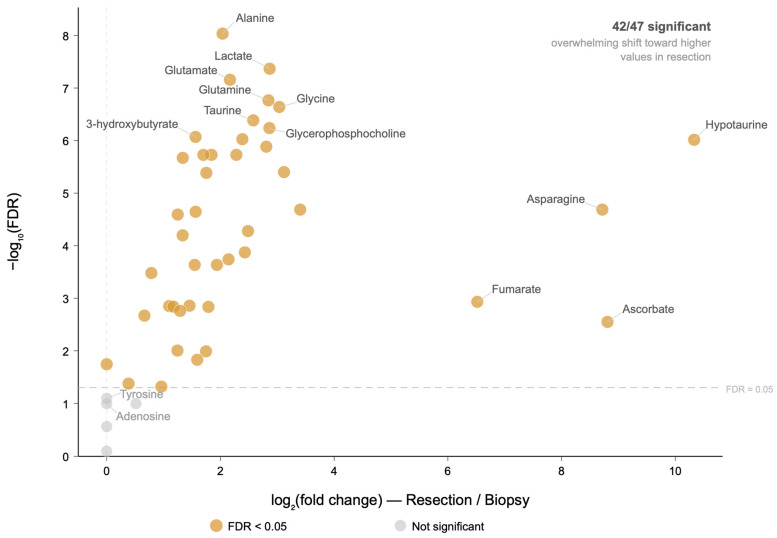
Volcano plot of metabolite-level comparisons between biopsy-only and resection-derived specimens (n = 99 de novo patients; 35 biopsy-only, 64 resection). Each point represents one of 47 HRMAS-quantified metabolites. The x-axis shows the log_2_ fold change (resection median/biopsy median) and the y-axis shows the −log_10_ of the Benjamini–Hochberg FDR-corrected *p*-value (Mann–Whitney U test). The horizontal dashed line indicates the significance threshold (FDR = 0.05). Orange points denote significant metabolites; gray points denote non-significant metabolites. Of the 42 metabolites reaching significance after FDR correction, 41 showed higher median concentrations in resection-derived samples, indicating an overwhelming shift toward higher metabolite levels in resection specimens. One metabolite (glutathione) reached significance due to a distributional difference despite identical group medians. Key metabolites are labeled. Log_2_ fold changes involving a zero group median were computed using a pseudocount. The complete list of all 47 metabolites is provided in Table S1.

To identify the metabolites underlying this global separation, we compared patient-level metabolite profiles between biopsy-only and resection-derived specimens. Of the 47 quantified metabolites, 42 remained significantly different after Benjamini–Hochberg correction (Table S1). Among these 42 metabolites, 41 showed higher median concentrations in resection-derived samples, indicating that the observed difference was not restricted to a small subset of analytes but instead reflected a broad shift across the measured metabolome (Figure 3). Glutathione was the only significant metabolite with a higher group median in resection, achieving significance due to a distributional difference despite identical medians in the two groups. The most strongly differentiated metabolites included alanine, lactate, glutamate, glutamine, glycine, taurine, glycerophosphocholine, 3-hydroxybutyrate, O-acetylcholine, and hypotaurine (Table 2). Complete metabolite-level results are provided in Table S1.

### 3.5. Pathway-Level Analysis Confirms a Global Metabolic Shift Associated with Surgical Sampling Context

**Figure 4:**
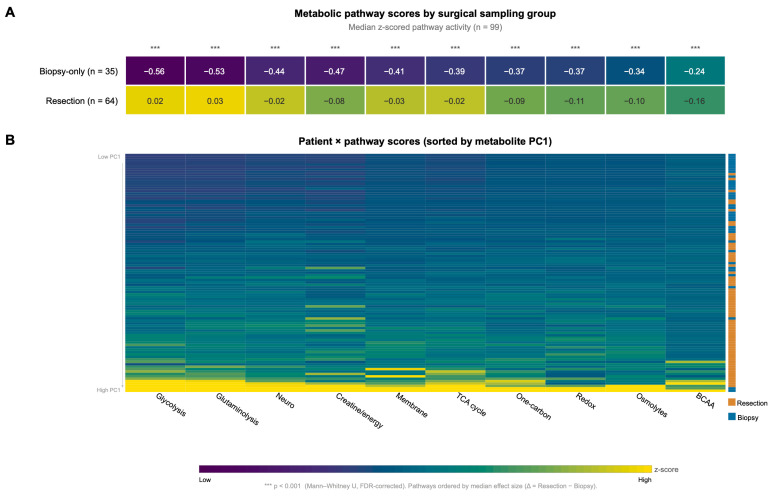
Metabolic pathway-level analysis. Forty-one assigned metabolites (6 unassigned; see Table S2) were grouped into 10 biologically defined metabolic pathways; pathway scores were computed as the mean of z-scored metabolite concentrations within each pathway. Pathways are ordered by decreasing median effect size (Δ = Resection median − Biopsy median). (**A**) Group-level summary heatmap showing median pathway z-scores for biopsy-only (n = 35) and resection (n = 64) specimens. Values are displayed within each cell. All 10 pathways showed significantly higher median scores in resection-derived samples after FDR correction (*** *p* < 0.001, Mann–Whitney U). Glutaminolysis and glycolysis exhibited the largest effect sizes. (**B**) Individual patient × pathway heatmap, with patients (rows) sorted by their metabolite-level PC1 score (low to high). The lateral color bar indicates surgical group membership (blue = biopsy, orange = resection). This visualization confirms that the pathway-level sampling effect is consistent across patients and is not driven by a subset of outliers.

To determine whether the group differences extended beyond individual metabolites, 41 assigned metabolites were grouped into 10 biologically defined metabolic pathways (Table S2), while 6 metabolites remained unassigned and were excluded from pathway-score calculations; pathway scores were then calculated as the mean of z-scored metabolite concentrations within each pathway. All 10 pathway scores were significantly higher in resection-derived samples after FDR correction (Figure 4A). The largest median differences were observed for glutaminolysis and glycolysis. At the individual-patient level, the pathway heatmap sorted by metabolite-level PC1 score showed that this pathway-level shift was broadly distributed across patients rather than driven by a limited number of outliers (Figure 4B).

### 3.6. Variance Partitioning and Confounder-Adjusted Analyses

To quantify the magnitude of the biopsy-versus-resection effect and account for baseline clinical and anatomical differences between groups (Table 1), we performed a formal variance partitioning of the patient-level z-scored metabolite matrix using PERMANOVA with 9999 permutations. In the unadjusted model, the surgical group alone explained 2.64% of the total metabolic variance (pseudo-F = 2.63, *p* = 0.026). After adjustment for age, WHO performance status ≥ 2, deep-seated location, midline/corpus callosum/deep-grey-nuclei involvement, multifocality, MGMT promoter methylation, and eloquent area involvement (by sequential residualization), the effect of the surgical group attenuated to 1.00% of residual variance and was no longer statistically significant (pseudo-F = 0.98, *p* = 0.39). The clinical covariates accounted for 5.36% of the total variance. These results indicate that, although the biopsy-versus-resection dichotomy is associated with a structured metabolic signature, the great majority of this association is statistically absorbed by the clinical and anatomical profile of the two groups.

Consistent with this variance-partitioning result, sensitivity analyses restricted to clinico-anatomically homogeneous subgroups progressively attenuated the metabolite-level effect (Table 3; Supplementary Figure S2). Excluding multifocal tumors reduced the number of FDR-significant metabolites from 42 to 29, and PC1 variance from 62.6% to 44.6%; excluding midline/corpus callosum tumors reduced them to 27 and 44.4%; excluding both yielded only 1 FDR-significant metabolite, although this final subgroup retained only 8 biopsy patients and was therefore substantially underpowered. PERMANOVA results showed the same pattern, becoming non-significant in all three restricted subgroups (all *p* ≥ 0.12).

We also tested whether the broad metabolite-level shift could be explained by a pure global-scaling (dilution) effect. We computed 15 canonical HRMAS-compatible metabolite ratios using creatine, total creatine (Cr + PCr), total choline (Cho + PCho + GPC), or NAA as denominators. Thirteen of the fifteen ratios remained significantly different between biopsy-only and resection-derived cases after FDR correction (all higher in resection; log_2_ fold changes +0.7 to +1.6), indicating that the effect cannot be reduced to a multiplicative scaling of all metabolites (Supplementary Figure S3A). Probabilistic Quotient Normalization (PQN), which specifically corrects for dilution, retained 16 of the 47 metabolites as significantly different (13 higher in resection), and the PERMANOVA R^2^ on PQN-normalized data was 4.09% (*p* = 0.0001), slightly higher than on the raw mass-normalized data (Supplementary Figure S3B). Importantly, the PQN dilution factors themselves differed significantly between groups (biopsy median 1.21 vs. resection median 1.04; Mann-Whitney *p* = 3.3 × 10^−5^), providing direct quantitative evidence that a tissue-composition component contributes to—but does not fully explain—the raw biopsy-versus-resection metabolic difference. Because per-spectrum tissue masses were comparable between groups (biopsy median 16.2 mg vs. resection median 16.65 mg, Mann-Whitney *p* = 0.13), this dilution-factor difference cannot be attributed to differential mass measurement.

### 3.7. Intratumoral Metabolic Heterogeneity Under Different Sampling Strategies

Intratumoral metabolic heterogeneity was examined in the 44 patients with ≥2 HRMAS spectra from the same surgical episode (18 biopsy-only, 26 resection; 107 intra-patient pairwise comparisons: 27 biopsy, 80 resection). The distribution of within-patient pairwise Euclidean distances was broader in the resection group than in the biopsy-only group (Supplementary Figure S1A). The patient-level heterogeneity score (mean intra-patient pairwise distance) was significantly higher in resection-derived cases (median 2.92 vs. 1.08; Mann–Whitney *p* = 0.002; Supplementary Figure S1B).

Because the number of spectra per patient differed between groups (biopsy: 17/18 patients with exactly 2 spectra; resection: range 2–7, median 2, mean 2.77; Mann-Whitney *p* = 0.008; Supplementary Figure S1C), we performed two sensitivity analyses to test whether the heterogeneity difference was confounded by this spectral-count asymmetry. First, within the multi-sampled cohort, the heterogeneity score correlated only weakly with the number of spectra per patient (Spearman ρ = 0.22, *p* = 0.16 overall; within-group ρ = 0.02 in biopsy and 0.06 in resection). Second, in an n-matched sensitivity analysis restricted to patients with exactly 2 spectra (n = 31; 17 biopsy, 14 resection), the heterogeneity difference was preserved (biopsy median 0.90 vs. resection median 2.82; Mann-Whitney *p* = 0.020). Together, these analyses indicate that the spectral-count difference between groups, although real, does not fully account for the observed heterogeneity difference.

Exploratory univariable Cox analyses identified no metabolite significantly associated with overall survival after FDR correction (complete results in Supplementary Table S3).

## 4. Discussion

In this study, we found that the apparent metabolic difference between biopsy-only and resection-derived HRMAS spectra in newly diagnosed IDH-wild-type glioblastoma is largely a composite signal. The surgical group alone explained only a modest share of the total patient-level metabolic variance (2.6%, *p* = 0.026). This share was no longer statistically significant after adjustment for age, WHO performance status, deep-seated location, midline involvement, multifocality, MGMT methylation, and eloquent area involvement (adjusted R^2^ = 1.0%, adjusted *p* = 0.39). Sensitivity analyses restricted to clinico-anatomically homogeneous subgroups progressively attenuated the metabolite-level effect, from 42 of 47 FDR-significant metabolites in the full cohort to 1 of 47 when both multifocal and midline tumors were excluded. This pattern indicates that the biopsy-versus-resection dichotomy captures, to a substantial extent, the clinical and anatomical profile of patients who preferentially undergo each procedure, rather than an intrinsic technical effect of the procedure itself. This interpretation is consistent with prior HRMAS studies showing that metabolic profiles in glioblastoma are closely linked to local tissue composition and intratumoral microheterogeneity, and with more recent spatially-resolved studies showing region-specific metabolic programs across distinct tumor compartments.

A plausible biological substrate for the residual metabolic shift is the difference in tissue composition between the two sampling contexts. Bulk resection specimens typically contain a higher proportion of viable tumor cells and a lower proportion of interstitial edema and non-tumoral brain than narrow stereotactic biopsies, particularly those performed on deep-seated or infiltrative tumors. Quantitatively, Probabilistic Quotient Normalization recovered significantly different dilution factors between the two groups (biopsy median 1.21 vs. resection median 1.04, *p* = 3.3 × 10^−5^), consistent with such a tissue-composition effect. Importantly, because per-spectrum tissue masses were comparable between groups (biopsy median 16.2 mg vs. resection median 16.65 mg, *p* = 0.13), this dilution-factor difference cannot be attributed to differential mass measurement and is best interpreted as reflecting a genuine tissue-composition difference. After explicit correction for this dilution component, 16 of 47 metabolites and 13 of 15 canonical metabolite ratios remained significantly different, indicating that tissue scaling alone does not fully account for the observed signal. These observations are consistent with the metabolic correlation patterns reported across the multi-center eTumor HRMAS dataset, which spans 132 glioblastomas and shows preserved metabolite-metabolite correlations across heterogeneous sampling contexts [14]. The biopsy-versus-resection effect is therefore best understood as a composite of (i) clinico-anatomical selection (the largest quantitative contributor), (ii) tissue composition and dilution (a non-trivial contributor), and (iii) a residual compositional difference that likely reflects genuine between-region metabolic differences

This structured sampling effect is biologically plausible. The present data do not indicate that the surgical act itself directly modifies tumor metabolite levels; rather, they show that the HRMAS profile recovered from tissue is strongly conditioned by the overall sampling context. Stereotactic biopsy is typically performed in tumors that are deep-seated, multifocal, poorly accessible, or located in eloquent or midline regions. It therefore captures a narrower and more constrained tissue fragment. By contrast, resection-derived material is obtained from a broader operative field. It is more likely to include spatially distinct tumor regions with differing proportions of viable tumor, infiltrative tissue, treatment-naïve stroma, necrotic components, and adjacent brain. In that setting, the biopsy-versus-resection variable should not be considered a simple surgical descriptor, but rather a major biological and pre-analytical determinant of the measured metabolomic profile. This view is in line with the neurosurgical literature on surgical indications and accessibility, as well as with recent recommendations emphasizing the importance of tissue location and sampling procedures for downstream biological analyses in diffuse gliomas [15,16,17].

The metabolite-level results support this interpretation particularly strongly. After correction for multiple testing, most quantified metabolites differed significantly between groups, and almost all significant metabolites showed higher median levels in resection-derived samples. This pattern argues against an isolated pathway-specific phenomenon and instead suggests a global shift in the measured tissue metabolome associated with sampling context. The pathway-level analysis further reinforced this point: all 10 predefined pathway scores were significantly higher in resection-derived specimens, and the patient-level heatmap showed that this effect was broadly distributed across the cohort rather than driven by a few extreme cases. Taken together, these findings suggest that sampling context shapes not only single-metabolite measurements but also the higher-order metabolic structure captured by HRMAS profiling. More recent tissue-based HRMAS work integrating machine-learning frameworks has identified N-acetylaspartate, glutamate, and glutamine as the most discriminative metabolites for glioma grade classification [18], findings that broadly align with the metabolite-level shifts observed in our cohort between biopsy-only and resection-derived samples.

Our heterogeneity analysis leads to the same conclusion. Multi-sampled resection cases showed greater within-patient metabolic dispersion than biopsy-only cases, as expected if a broader surgical field captures a wider range of intratumoral states. Importantly, the lower heterogeneity observed in biopsy-only cases should not be interpreted as evidence of true biological homogeneity. Rather, it likely reflects the narrower sampling window imposed by stereotactic targeting. In that sense, the heterogeneity score used here should be understood as a measure of observed heterogeneity under a given sampling strategy, not as a direct estimate of the tumor’s full metabolic complexity. This interpretation is consistent with early HRMAS work showing marked intratumoral metabolic variation across specimens from the same glioblastoma, and with more recent spatial and multi-omics studies showing that distinct biological states can coexist across different tumor regions, including in samples obtained through needle-based approaches [4,8,19,20].

By contrast, exploratory survival analyses did not identify any robust metabolite-level prognostic signal after correction for multiple testing. This negative result should be interpreted cautiously. It does not imply that tumor metabolism is clinically irrelevant in glioblastoma, but rather underscores how difficult it remains to derive stable prognostic biomarkers from tissue-based metabolomic data in relatively small cohorts characterized by high dimensionality, marked spatial heterogeneity, and clinically non-equivalent sampling conditions. Previous studies have reported associations between metabolite patterns and survival in diffuse glioma and glioblastoma across both tissue and biofluid platforms. Still, these findings remain heterogeneous across cohorts, sampled regions, analytical platforms, and modeling strategies [2,21,22,23,24]. No metabolomics-derived biomarker is currently used in routine practice for prognostication in glioblastoma.

Several limitations should be acknowledged. First, this was a single-centre retrospective analysis of a prospectively collected tissue program, and the biopsy-only and resection-derived groups were not matched for surgical indication. Accordingly, the observed biopsy-versus-resection signal should be interpreted as a composite sampling-context effect, integrating both the surgical access window and the clinicoradiological factors that determine surgical indication, rather than as a purely technical effect. In glioblastoma, the decision to perform a biopsy rather than a resection is inherently shaped by resectability, anatomical accessibility, eloquence, and clinical status, which introduces unavoidable selection bias when comparing biological readouts across surgical contexts [13,14]. Second, structured per-spectrum annotation of contrast-enhancing versus FLAIR localization was not systematically available in our database across the 2019–2024 window, precluding a formal within-region comparison; regional sampling is very likely to contribute to the biopsy-versus-resection effect reported here, since stereotactic biopsy preferentially targets the contrast-enhancing edge of deep or eloquent tumors, whereas resection samples a broader territory including more tumor core. This limitation should be addressed prospectively using spatially annotated, neuronavigation-tagged sampling and emerging spatially localized HRMAS acquisition schemes. However, within-sample spatial resolution [17,20,25]. Third, systematic paired histopathological annotation at exact HRMAS sampling coordinates was not available because routine tissue banking in our program flash-froze the entire fragment for HRMAS analysis; mirror formalin-fixed samples taken at the precise HRMAS coordinates were available only for a minority of cases, which prevented quantitative integration of cellularity and necrosis per spectrum. Fourth, pipeline stability across the 2019–2024 window and between-group spectral-quality homogeneity were documented primarily through visual quality control by the HRMAS operator rather than through systematic numerical group-level summaries such as signal-to-noise ratio or linewidth. However, a representative subset of spectra was re-examined quantitatively and did not suggest a between-group imbalance; per-spectrum acquisition metrics could not be retrospectively extracted systematically across the full archived dataset, and such metrics should therefore be reported prospectively in future HRMAS studies. Finally, the pathway-level analysis should be interpreted as a structured data-reduction approach rather than a direct measurement of metabolic flux, and the exploratory Cox survival analyses (Supplementary Table S3) remain constrained by sample size and multiple-testing burden.

Taken together, our results indicate that the main translational challenge in tissue-based metabolomics of glioblastoma is not only analytical sensitivity, but also control of sampling context. HRMAS remains a powerful technique for extracting biologically meaningful metabolic information from intact tumor tissue while preserving histological interpretability. Still, its value depends critically on how, where, and under which surgical constraints specimens are obtained. Future metabolomic studies in glioblastoma should therefore avoid treating biopsy-only and resection-derived specimens as interchangeable inputs and should incorporate spatially informed sampling frameworks whenever possible. Notably, the progressive attenuation observed across anatomically restricted subgroups suggests that deep-seated, midline, and multifocal IDH-wild-type glioblastomas may themselves carry a distinct HRMAS metabolic signature, a hypothesis that warrants dedicated investigation in an adequately powered cohort and is beyond the scope of the present methodological report. From this perspective, the present work does not merely document a sampling bias; it defines a methodological boundary condition for the development of robust and biologically interpretable tissue metabolomic biomarkers in glioblastoma [3,17,20].

## 5. Conclusions

HRMAS metabolomic profiling of newly diagnosed IDH-wild-type glioblastoma shows that the apparent metabolic difference between biopsy-only and resection-derived specimens is largely a composite sampling-context signal reflecting clinical and anatomical patient selection and tissue composition, with only a small residual share attributable to surgical access per se. Biopsy-only and resection-derived specimens should therefore not be considered metabolically interchangeable, and the extent of measured intratumoral metabolic heterogeneity is strongly conditioned by the sampling strategy.

Practically, the surgical sampling context should be explicitly reported for every tissue-based metabolomic study in glioblastoma, and pooled analyses across biopsy and resection specimens should either be avoided or accompanied by stratified analyses and adjustment for clinico-anatomical covariates. Prospective studies incorporating neuronavigation-tagged or MRI-registered intraoperative sampling will be essential to enable rigorous within-region comparisons and advance the translational development of tissue-based metabolomic biomarkers.

## Figures and Tables

**Table 1:** Clinical and tumor characteristics of the de novo IDH-wild-type glioblastoma cohort. Values are median [IQR] or n (%). *p*: Mann–Whitney U test for continuous variables, Fisher’s exact test for binary categorical variables, and chi-square test for categorical variables with > 2 levels, as appropriate. CC—corpus callosum; CE—contrast-enhancing; GTR—gross-total resection; NTR—near-total resection; STR—subtotal resection; WHO—World Health Organization. MGMT denominators reflect available data (6 missing). ‡ Chi-square test.

Variable	Overall (n/99)	Biopsy-Only (n = 35)	Resection (n = 64)	*p*
***Demographics***				
Age, years	63.7 [56.2–69.3]	63.5 [56.5–69.5]	63.8 [55.9–69.0]	0.626
Male sex	66 (66.7%)	21 (60.0%)	45 (70.3%)	0.373
WHO performance status ≥ 2	22 (22.2%)	13 (37.1%)	9 (14.1%)	0.012
***Tumor characteristics***				
**Tumor side**				<0.001 ‡
Left hemisphere	45 (45.5%)	16 (45.7%)	29 (45.3%)	
Right hemisphere	47 (47.5%)	12 (34.3%)	35 (54.7%)	
Bilateral	7 (7.1%)	7 (20.0%)	0 (0%)	
Deep-seated location	41 (41.4%)	30 (85.7%)	11 (17.2%)	<0.001
Eloquent area	58 (58.6%)	32 (91.4%)	26 (40.6%)	<0.001
Midline/CC/deep nuclei	24 (24.2%)	22 (62.9%)	2 (3.1%)	<0.001
Multifocal	27 (27.3%)	21 (60.0%)	6 (9.4%)	<0.001
Dominant hemisphere	54 (54.5%)	25 (71.4%)	29 (45.3%)	0.020
Max CE diameter				0.057 ‡
<3 cm	19 (19.2%)	9 (25.7%)	10 (15.6%)	
3–5 cm	23 (23.2%)	4 (11.4%)	19 (29.7%)	
>5 cm	57 (57.6%)	22 (62.9%)	35 (54.7%)	
***Molecular markers***				
MGMT methylated	45/93 (48.4%)	16/33 (48.5%)	29/60 (48.3%)	1.000
***Treatment and outcome***				
Stupp protocol	83 (83.8%)	24 (68.6%)	59 (92.2%)	0.004
Extent of resection (resection group only)	—	—	GTR 40/NTR 21/STR 3	—
Deaths	83 (83.8%)	29 (82.9%)	54 (84.4%)	1.000
Overall survival, days	513 [254–862]	266 [79–509]	620 [360–951]	<0.001

**Table 2:** Top 20 differentially quantified metabolites between biopsy-only and resection-derived IDH-wild-type glioblastoma specimens (n = 99 de novo patients; 35 biopsy-only, 64 resection). 42 of 47 metabolites reached significance after FDR correction (complete results in Table S1). Of these, 41 showed higher median concentrations in resection-derived samples; glutathione reached significance due to a distributional difference despite identical group medians. FC—fold change (resection/biopsy median); FDR—false discovery rate. †—Pseudocount used (median = 0 in ≥1 group); FC—not reported. Values expressed in nmol/mg.

Metabolite	Biopsy-Only Median [IQR]	Resection Median [IQR]	FC	*p*	FDR
Alanine	0.985 [0.456–1.38]	4.04 [2.82–6.29]	4.1	<0.001	9.2 × 10^−9^
Lactate	3.97 [2.45–8.80]	28.9 [19.0–41.0]	7.3	<0.001	4.3 × 10^−8^
Glutamate	2.81 [1.56–4.21]	12.6 [7.50–17.5]	4.5	<0.001	7.0 × 10^−8^
Glutamine	0.612 [0.302–1.74]	4.40 [2.41–5.99]	7.2	<0.001	1.7 × 10^−7^
Glycine	1.03 [0.490–1.94]	8.44 [4.00–15.4]	8.2	<0.001	2.3 × 10^−7^
Taurine	0.327 [0.184–0.725]	1.95 [1.04–3.26]	6.0	<0.001	4.1 × 10^−7^
Glycerophosphocholine	0.192 [0.0979–0.511]	1.40 [0.676–2.28]	7.3	<0.001	5.8 × 10^−7^
3-hydroxybutyrate	0.170 [0.0000–0.306]	0.501 [0.313–0.720]	3.0	<0.001	8.5 × 10^−7^
O-acetylcholine	0.0095 [0.0057–0.0164]	0.0495 [0.0281–0.0821]	5.2	<0.001	9.4 × 10^−7^
Hypotaurine	0.0000 [0.0000–0.295]	1.29 [0.557–3.00]	†	<0.001	9.6 × 10^−7^
Choline	0.336 [0.214–0.648]	2.35 [0.921–4.64]	7.0	<0.001	1.3 × 10^−6^
Proline	0.448 [0.135–0.766]	1.61 [0.945–3.11]	3.6	<0.001	1.9 × 10^−6^
Phosphocholine	0.495 [0.181–0.710]	1.61 [0.810–2.49]	3.2	<0.001	1.9 × 10^−6^
Threonine	0.392 [0.229–0.843]	1.91 [1.18–2.91]	4.9	<0.001	1.9 × 10^−6^
Leucine	0.460 [0.286–0.676]	1.16 [0.775–1.52]	2.5	<0.001	2.1 × 10^−6^
Ethanolamine	0.242 [0.0000–0.665]	2.10 [0.830–3.77]	8.7	<0.001	4.0 × 10^−6^
Serine	0.942 [0.410–1.65]	3.17 [2.44–4.15]	3.4	<0.001	4.1 × 10^−6^
Allocystathionine	0.125 [0.0000–0.519]	1.32 [0.685–2.09]	11	<0.001	2.1 × 10^−5^
Asparagine	0.0000 [0.0000–0.0800]	0.420 [0.0000–0.702]	†	<0.001	2.1 × 10^−5^
Succinate	0.188 [0.0277–0.309]	0.555 [0.308–0.921]	3.0	<0.001	2.3 × 10^−5^

**Table 3:** Variance partitioning and clinico-anatomical sensitivity analyses. * Covariates: age, WHO PS ≥ 2, deep-seated location, midline/CC/NGC involvement, multifocality, MGMT, eloquent area. Clinical covariates alone absorb 5.36% of total metabolic variance.

Analysis	n	n bio/res	# metab. sig.	PC1 var %	PC1 *p*	PERMANOVA R^2^	PERMANOVA *p*
Full cohort—unadjusted	99	35/64	42/47	62.6%	3.3 × 10^−8^	2.64%	0.026
Full cohort—adjusted *	99	35/64	—	—	—	1.00% (resid.)	0.39
Excluding multifocal	72	14/58	29/47	44.6%	3.3 × 10^−4^	2.30%	0.125
Excluding midline/CC/NGC	75	13/62	27/47	44.4%	4.3 × 10^−4^	1.89%	0.171
Excluding multifocal and midline	65	8/57	1/47	44.0%	0.013	1.36%	0.428

## Data Availability

The anonymized patient-level metabolomic dataset and analysis code supporting the findings of this study are available from the corresponding author upon reasonable request. Restrictions apply to clinical data, which were used under institutional ethical approval and are not publicly available due to patient privacy considerations.

## Supplementary Materials

The following supporting information can be downloaded at: https://www.mdpi.com/article/10.3390/metabo16050296/s1, Table S1: Complete comparison of all 47 HRMAS metabolites between biopsy-only and resection-derived specimens; Table S2: Assignment of the 47 HRMAS-quantified metabolites to 10 biologically defined metabolic pathways; Table S3: Univariable Cox proportional hazards analysis of individual metabolites and overall survival; Figure S1: Intratumoral metabolic heterogeneity and sampling intensity in multi-sampled patients; Figure S2: Sensitivity analyses across clinico-anatomically restricted subgroups; Figure S3: Sensitivity analyses for tissue-scaling effects (canonical metabolite ratios and PQN normalization).

## Abbreviations

The following abbreviations are used in this manuscript:

BCAA	Branched-chain amino acids
CC	Corpus callosum
CE	Contrast-enhancing
CNS	Central nervous system
CPMG	Carr–Purcell–Meiboom–Gill (pulse sequence)
D_2_O	Deuterium oxide
ERETIC	Electronic reference to access in vivo concentrations
FC	Fold change
FDR	False discovery rate
FLAIR	Fluid-attenuated inversion recovery
GABA	γ-Aminobutyric acid
GTR	Gross-total resection
HR	Hazard ratio
HRMAS	High-resolution magic angle spinning
IDH	Isocitrate dehydrogenase
IQR	Interquartile range
IRB	Institutional review board
MGMT	O^6^-methylguanine-DNA methyltransferase
MRI	Magnetic resonance imaging
NMR	Nuclear magnetic resonance
NTR	Near-total resection
OS	Overall survival
PC1	First principal component
PCA	Principal component analysis
ROSA	Robotized Stereotactic Assistant
SD	Standard deviation
STR	Subtotal resection
TCA	Tricarboxylic acid (cycle)
WHO	World Health Organization
