# Supplementary material for: High-Resolution Magic Angle Spinning Metabolomic Profiling of IDH-Wild-Type Glioblastoma Reveals a Composite Surgical Sampling Signature Shaped by Clinical and Anatomical Tumor Features
Source: Metabolites. 2026 Apr 27;16(5):296. doi: 10.3390/metabo16050296 (PMC13208751; doi:10.3390/metabo16050296)
Supplement: Supplementary file 1 [file metabolites-16-00296-s001.zip › Figure S1.pdf]

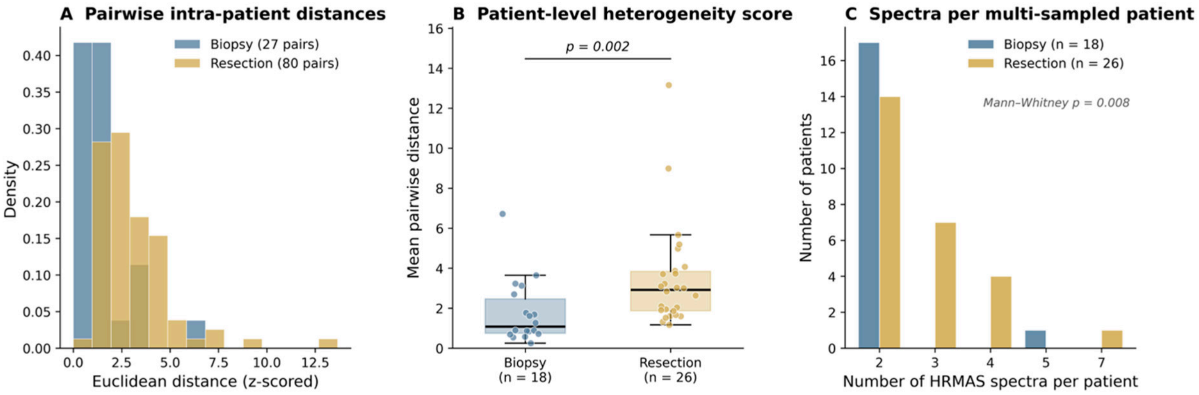

Figure S1. *Intratumoral metabolic heterogeneity and sampling intensity in multi-sampled patients (n = 44; 18 biopsy-only, 26 resection). (A) Distribution of pairwise Euclidean distances on z-scored spectrum-level profiles (biopsy: 27 pairs; resection: 80 pairs). (B) Patient-level heterogeneity scores (mean intra-patient pairwise distance). Resection specimens showed greater heterogeneity than biopsy specimens (p = 0.002, Mann–Whitney U). Box: median and IQR; whiskers: 1.5 × IQR; dots: individual patients. (C) Distribution of HRMAS spectra per patient. Biopsy cases were predominantly sampled twice (17/18), whereas resection cases ranged from 2 to 7 spectra (p = 0.008). Sensitivity analyses confirmed the heterogeneity difference was not driven by spectral count (Spearman ρ = 0.22, p = 0.16; n-matched analysis on patients with exactly 2 spectra: 17 biopsy vs 14 resection, p = 0.020).*
