# Supplementary material for: High-Resolution Magic Angle Spinning Metabolomic Profiling of IDH-Wild-Type Glioblastoma Reveals a Composite Surgical Sampling Signature Shaped by Clinical and Anatomical Tumor Features
Source: Metabolites. 2026 Apr 27;16(5):296. doi: 10.3390/metabo16050296 (PMC13208751; doi:10.3390/metabo16050296)

Figure S2. *Volcano plots of metabolite-wise log_2 fold change (resection/biopsy) versus −log_{10}(FDR) across four clinico-anatomically restricted cohorts, for all 47 HRMAS-quantified metabolites. Orange: FDR < 0.05; grey: non-significant; dashed line: FDR = 0.05; triangles: values beyond displayed range. From left to right: full cohort (n = 99; 42/47 significant); excluding multifocal (n = 72; 29/47); excluding midline/CC/deep grey nuclei (n = 75; 27/47); excluding both (n = 65; 1/47). The progressive attenuation indicates that the metabolite-level biopsy-vs-resection signal is largely explained by the clinico-anatomical profile of tumors preferentially undergoing each surgical procedure.*

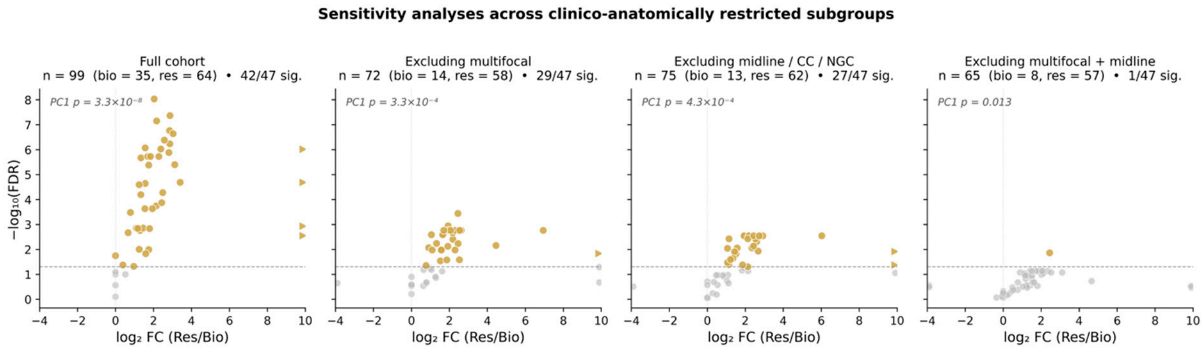

Supplement: Supplementary file 1 [file metabolites-16-00296-s001.zip › Figure S2.pdf]
