# Supplementary material for: High-Resolution Magic Angle Spinning Metabolomic Profiling of IDH-Wild-Type Glioblastoma Reveals a Composite Surgical Sampling Signature Shaped by Clinical and Anatomical Tumor Features
Source: Metabolites. 2026 Apr 27;16(5):296. doi: 10.3390/metabo16050296 (PMC13208751; doi:10.3390/metabo16050296)

Figure S3. *Sensitivity analyses for tissue-scaling effects. (**A**) Log$_2$ fold changes (resection/biopsy) for 15 canonical HRMAS metabolite ratios using creatine (Cr), total-creatine (tCr), total-choline (tCho), or N-acetylaspartate (NAA) as denominators [14]. Orange: FDR-significant; grey: non-significant. Thirteen of 15 ratios differed significantly between groups, indicating that the compositional difference is not reducible to a single-metabolite normalisation. (**B**) Effect of Probabilistic Quotient Normalisation (PQN) on metabolite-wise log$_2$ fold changes. Each point represents one of 47 metabolites; orange: significant after PQN; grey: loses significance. After PQN correction, 16/47 metabolites remained significantly different, indicating a residual compositional shift beyond pure scaling. PQN dilution factors differed between groups (biopsy median 1.21 vs resection 1.04; $p = 3\times10^{-5}$).*

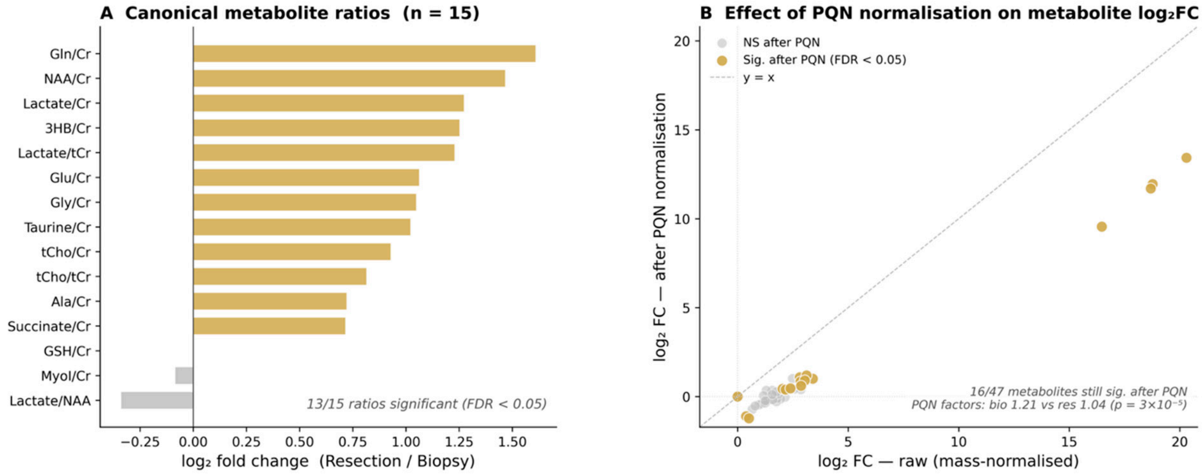

Supplement: Supplementary file 1 [file metabolites-16-00296-s001.zip › Figure S3.pdf]
