# Supplementary material for: High-Resolution Magic Angle Spinning Metabolomic Profiling of IDH-Wild-Type Glioblastoma Reveals a Composite Surgical Sampling Signature Shaped by Clinical and Anatomical Tumor Features
Source: Metabolites. 2026 Apr 27;16(5):296. doi: 10.3390/metabo16050296 (PMC13208751; doi:10.3390/metabo16050296)
Supplement: Supplementary file 1 [file metabolites-16-00296-s001.zip › TableS1.pdf]

Table S1. Complete comparison of all 47 HRMAS metabolites (n = 99 de novo patients; 35 biopsy-only, 64 resection).

Metabolite	Biopsy-only Median [IQR]	Resection Median [IQR]	FC	p	FDR
Alanine*	0.985 [0.456–1.38]	4.04 [2.82–6.29]	4.1	<0.001	9.2e-9
Lactate*	3.97 [2.45–8.80]	28.9 [19.0–41.0]	7.3	<0.001	4.3e-8
Glutamate*	2.81 [1.56–4.21]	12.6 [7.50–17.5]	4.5	<0.001	7.0e-8
Glutamine*	0.612 [0.302–1.74]	4.40 [2.41–5.99]	7.2	<0.001	1.7e-7
Glycine*	1.03 [0.490–1.94]	8.44 [4.00–15.4]	8.2	<0.001	2.3e-7
Taurine*	0.327 [0.184–0.725]	1.95 [1.04–3.26]	6.0	<0.001	4.1e-7
Glycerophosphocholine*	0.192 [0.0979–0.511]	1.40 [0.676–2.28]	7.3	<0.001	5.8e-7
3-hydroxybutyrate*	0.170 [0.0000–0.306]	0.501 [0.313–0.720]	3.0	<0.001	8.5e-7
O-acetylcholine*	0.0095 [0.0057–0.0164]	0.0495 [0.0281–0.0821]	5.2	<0.001	9.4e-7
Hypotaurine*	0.0000 [0.0000–0.295]	1.29 [0.557–3.00]	†	<0.001	9.6e-7
Choline*	0.336 [0.214–0.648]	2.35 [0.921–4.64]	7.0	<0.001	1.3e-6
Proline*	0.448 [0.135–0.766]	1.61 [0.945–3.11]	3.6	<0.001	1.9e-6
Phosphocholine*	0.495 [0.181–0.710]	1.61 [0.810–2.49]	3.2	<0.001	1.9e-6
Threonine*	0.392 [0.229–0.843]	1.91 [1.18–2.91]	4.9	<0.001	1.9e-6
Leucine*	0.460 [0.286–0.676]	1.16 [0.775–1.52]	2.5	<0.001	2.1e-6
Ethanolamine*	0.242 [0.0000–0.665]	2.10 [0.830–3.77]	8.7	<0.001	4.0e-6
Serine*	0.942 [0.410–1.65]	3.17 [2.44–4.15]	3.4	<0.001	4.1e-6
Allocystathionine*	0.125 [0.0000–0.519]	1.32 [0.685–2.09]	11	<0.001	2.1e-5
Asparagine*	0.0000 [0.0000–0.0800]	0.420 [0.0000–0.702]	†	<0.001	2.1e-5
Succinate*	0.188 [0.0277–0.309]	0.555 [0.308–0.921]	3.0	<0.001	2.3e-5
Valine*	0.293 [0.161–0.497]	0.696 [0.449–0.940]	2.4	<0.001	2.6e-5
N-acetylaspartate*	0.130 [0.0000–0.459]	0.729 [0.393–1.60]	5.6	<0.001	5.3e-5
Arginine*	0.246 [0.0539–0.331]	0.620 [0.321–0.912]	2.5	<0.001	6.4e-5
Phosphocreatine*	0.192 [0.0161–0.754]	1.03 [0.466–1.78]	5.4	<0.001	1.3e-4
2-hydroxyglutarate*	0.267 [0.0000–0.567]	1.18 [0.331–1.76]	4.4	<0.001	1.8e-4
Betaine*	0.0798 [0.0225–0.177]	0.233 [0.122–0.421]	2.9	<0.001	2.3e-4
Glycerol*	0.761 [0.439–1.26]	2.92 [1.31–4.41]	3.8	<0.001	2.3e-4
Lysine*	0.401 [0.172–0.508]	0.692 [0.408–1.08]	1.7	<0.001	3.3e-4
Fumarate*	0.0000 [0.0000–0.0000]	0.0906 [0.0000–0.212]	†	<0.001	0.001
GABA*	0.294 [0.0314–0.667]	0.808 [0.343–1.62]	2.7	<0.001	0.001
Creatine*	1.31 [0.550–2.91]	2.81 [1.97–5.91]	2.1	<0.001	0.001
N-acetyl-lysine*	0.0401 [0.0000–0.0654]	0.0905 [0.0333–0.156]	2.3	<0.001	0.001
Ornithine*	0.154 [0.0000–0.460]	0.532 [0.282–0.658]	3.5	0.001	0.001
Aspartate*	1.10 [0.0000–2.73]	2.68 [1.52–4.61]	2.4	0.001	0.002
Isoleucine*	0.121 [0.0505–0.171]	0.191 [0.129–0.272]	1.6	0.002	0.002
Ascorbate*	0.0000 [0.0000–0.0571]	0.447 [0.0000–0.893]	†	0.002	0.003
Myo-inositol*	0.819 [0.349–2.35]	1.94 [1.03–4.40]	2.4	0.008	0.010
Methionine*	0.0407 [0.0000–0.105]	0.137 [0.0421–0.217]	3.4	0.008	0.010
Scyllo-inositol*	0.0257 [0.0066–0.0923]	0.0776 [0.0386–0.172]	3.0	0.012	0.015
Glutathione*	0.0000 [0.0000–0.0000]	0.0000 [0.0000–0.778]	†	0.015	0.018
Acetate*	0.793 [0.276–1.04]	1.04 [0.544–1.22]	1.3	0.037	0.042
Glucose*	2.17 [0.450–5.01]	4.23 [1.87–7.00]	1.9	0.043	0.048
Tyrosine	0.0000 [0.0000–0.0000]	0.0000 [0.0000–0.203]	†	0.073	0.080
Adenosine	0.0000 [0.0000–0.230]	0.0000 [0.0000–0.112]	†	0.096	0.101
Ethanol	0.206 [0.0676–0.355]	0.295 [0.165–0.444]	1.4	0.097	0.101
Phenylalanine	0.0000 [0.0000–0.0763]	0.0000 [0.0000–0.239]	†	0.268	0.273
Formate	0.0000 [0.0000–0.126]	0.0000 [0.0000–0.158]	†	0.795	0.795

** FDR < 0.05. † Pseudocount used (median = 0 in ≥ 1 group); FC not reported. Of the 42 significant metabolites, 41 showed higher median values in resection-derived samples; glutathione was significant due to a distributional shift despite identical group medians. 0.000 = below HRMAS detection threshold. Values expressed in nmol/mg.*
