# Supplementary material for: High-Resolution Magic Angle Spinning Metabolomic Profiling of IDH-Wild-Type Glioblastoma Reveals a Composite Surgical Sampling Signature Shaped by Clinical and Anatomical Tumor Features
Source: Metabolites. 2026 Apr 27;16(5):296. doi: 10.3390/metabo16050296 (PMC13208751; doi:10.3390/metabo16050296)
Supplement: Supplementary file 1 [file metabolites-16-00296-s001.zip › TableS2.pdf]

**Table S2.** Assignment of the 47 HRMAS-quantified metabolites to 10 biologically defined metabolic pathways, with 6 metabolites left unassigned and excluded from pathway-score calculations.

| Metabolic pathway | Constituent metabolites | n |
|---|---|---|
| Glycolysis | Lactate, Glucose, Alanine, Glycerol, 3-hydroxybutyrate, Acetate | 6 |
| Glutaminolysis | Glutamate, Glutamine, Asparagine, Aspartate, Proline, 2-hydroxyglutarate | 6 |
| Neuro (neuronal markers) | N-acetylaspartate, GABA, Adenosine, O-acetylcholine | 4 |
| Creatine / energy | Creatine, Phosphocreatine | 2 |
| Membrane (phospholipid) | Choline, Phosphocholine, Glycerophosphocholine, Ethanolamine | 4 |
| TCA cycle | Succinate, Fumarate | 2 |
| One-carbon | Glycine, Serine, Methionine, Formate, Threonine, Betaine | 6 |
| Redox | Glutathione, Ascorbate, Hypotaurine, Taurine, Allocystathionine | 5 |
| Osmolytes | Myo-inositol, Scyllo-inositol, Ethanol | 3 |
| BCAA | Leucine, Isoleucine, Valine | 3 |
| Not assigned* | Arginine, Lysine, N-acetyl-lysine, Ornithine, Phenylalanine, Tyrosine | 6 |

* *Not included in pathway-score calculations. BCAA = branched-chain amino acids; TCA = tricarboxylic acid.*
