# Supplementary material for: High-Resolution Magic Angle Spinning Metabolomic Profiling of IDH-Wild-Type Glioblastoma Reveals a Composite Surgical Sampling Signature Shaped by Clinical and Anatomical Tumor Features
Source: Metabolites. 2026 Apr 27;16(5):296. doi: 10.3390/metabo16050296 (PMC13208751; doi:10.3390/metabo16050296)
Supplement: Supplementary file 1 [file metabolites-16-00296-s001.zip › TableS3.pdf]

Table S3. Univariable Cox proportional hazards analysis of individual metabolites and overall survival (n = 99 de novo IDH-wild-type glioblastoma; 83 events).

Metabolite	HR per +1 SD	95% CI	p	FDR
Adenosine	1.42	1.11–1.82	0.005	0.147
Lysine	1.36	1.06–1.75	0.016	0.147
Ascorbate	1.39	1.05–1.84	0.020	0.147
Phosphocholine	1.36	1.05–1.76	0.020	0.147
Proline	1.35	1.05–1.74	0.021	0.147
Glycerol	1.34	1.04–1.72	0.024	0.147
Valine	1.32	1.01–1.73	0.043	0.188
Glycerophosphocholine	1.35	1.00–1.83	0.049	0.188
Taurine	1.34	0.99–1.81	0.056	0.188
Ornithine	1.30	0.99–1.70	0.060	0.188
Phosphocreatine	1.31	0.98–1.74	0.065	0.188
Succinate	1.29	0.98–1.70	0.067	0.188
Leucine	1.28	0.98–1.68	0.068	0.188
3-hydroxybutyrate	0.84	0.66–1.07	0.167	0.429
N-acetylaspartate	1.23	0.86–1.74	0.256	0.518
Threonine	1.20	0.88–1.64	0.258	0.518
Aspartate	1.13	0.92–1.38	0.262	0.518
Choline	1.14	0.91–1.43	0.265	0.518
Serine	1.17	0.88–1.54	0.278	0.518
GABA	1.15	0.89–1.47	0.288	0.518
Myo-inositol	1.12	0.89–1.43	0.339	0.581
Acetate	1.09	0.90–1.32	0.381	0.623
Ethanolamine	1.09	0.88–1.34	0.448	0.688
Betaine	1.13	0.82–1.54	0.458	0.688
Glucose	1.07	0.86–1.33	0.528	0.743
Alanine	1.07	0.85–1.35	0.540	0.743
Glycine	1.07	0.85–1.34	0.568	0.743
Glutamate	1.06	0.86–1.29	0.592	0.743
Arginine	1.05	0.87–1.28	0.599	0.743
Allocystathionine	0.95	0.77–1.17	0.632	0.759
2-hydroxyglutarate	1.04	0.83–1.29	0.745	0.834
Glutamine	0.96	0.75–1.23	0.753	0.834
Hypotaurine	1.04	0.79–1.37	0.764	0.834
Lactate	0.98	0.78–1.23	0.851	0.896
Asparagine	1.02	0.80–1.31	0.872	0.896
Creatine	1.02	0.81–1.27	0.896	0.896
Ethanol	—	—	—	—
Formate	—	—	—	—
Fumarate	—	—	—	—
Glutathione	—	—	—	—
Isoleucine	—	—	—	—
Methionine	—	—	—	—
N-acetyl-lysine	—	—	—	—
O-acetylcholine	—	—	—	—
Phenylalanine	—	—	—	—
Scyllo-inositol	—	—	—	—
Tyrosine	—	—	—	—

HR = hazard ratio per +1 standard deviation increase (z-score standardized). FDR: Benjamini–Hochberg correction on 36 converged models. No metabolite reached significance after correction (minimum FDR = 0.147, Adenosine). — = model did not converge (insufficient variance in predictor). Sorted by ascending p-value.
